# The epitope of the VP1 protein of porcine parvovirus

**DOI:** 10.1186/1743-422X-7-161

**Published:** 2010-07-16

**Authors:** Hong-ling Xie, Zhao Wang, Shang-jin Cui, Chao-fan Zhang, Yu-dong Cui

**Affiliations:** 1 Division of Swine Infection Disease, State Key Laboratory of Veterinary Biotechnology, Harbin Veterinary Research Institute of Chinese Academy of Agricultural Sciences, Harbin 150001, China; 2 College of Animal Science and Technology, Hei long Jiang August First Land Reclamation University, Daqing 163319, China; 3 Qingdao Agricultural University, Qingdao 266109, China

## Abstract

Porcine parvovirus (PPV) is the major causative agent in a syndrome of reproductive failure in swine. Much has been learned about the structure and function of PPV in recent years, but nothing is known about the epitopes of the structural protein VP1, which is an important antigen of PPV. In this study, the monoclonal antibody C4 against VP1 of PPV was prepared and was used to biopan a 12-mer phage peptide library three times. The selected phage clones were identified by ELISA and then sequencing. The amino acid sequences detected by phage display were analyzed, and a mimic immuno-dominant epitope was identified. The epitope of VP1 is located in the N-terminal and contains the role amino acid sequence R-K-R. Immunization of mice indicated that the phage-displayed peptide induces antibodies against PPV. This study shows that peptide mimotopes have potential as alternatives to the complex antigens currently used for diagnosis of PPV infection or for development of vaccines.

## Introduction

Porcine parvovirus (PPV), which was first isolated from sows in Germany by Mayr et al. [1] and which belongs to the genus Parvovirus in the family Parvoviridae, is the major causative virus in a syndrome of reproductive failure in swine. This syndrome is referred to as SEDI and includes stillbirths, mummified fetuses, early embryonic death, and infertility [2]. Recent studies indicate that, in addition to inducing reproductive failure, PPV also causes dermatitis, diarrhea, and respiratory system disease [3-10].

PPV is composed of structural protein and non-structural protein, and the structural protein is the virus's main immunological antigen. Using SDS-PAGE, Moliter et al. [11] identified three kinds of structural proteins: VP1, VP2, and VP3. All three of these proteins can induce hemagglutination inhibition (HI) antibodies and neutralization antibodies in rabbits; induction of HI antibodies was greatest with VP3, intermediate with VP2, and least with VP1.

VP1 has an important biological function for PPV: its intranuclear signal sequence, which is similar to that of the VP1 proteins of Simian virus 40 and human papillomavirus [12], is very important for PPV positioning within the host nucleus. The N-terminal of VP1 is rich in alkaline amino acids, which enhance binding with host DNA to stabilize viral single-strand genome DNA; the binding is required for the initiation of viral DNA reproduction and for the packaging of the viral genome [13].

The structural proteins of a related virus, canine parvovirus (CPV), have three antigen epitope regions In the case of CPV, the epitope located in region B1 possesses good antigenicity and induces the host to produce neutralization antibody [14,15]. Unfortunately, such information about antigen epitope is unavailable for PPV.

Phage display is a powerful tool for the study of the interaction between antigen and antibody. This molecular biology technique can directly select simulated antigen epitopes that can combine to antibody in random protein banks. Before phage display can be used, the sequence of amino acids in the epitope must be determined [16]. Using monoclonal antibodies, we selected one peptide that mimics the VP1 epitope of PPV from a phage-displayed random peptide library. To assess its diagnostic value, we screened a panel of 12 individual PPV sera for their reactivity with the peptides alone. The information obtained from this screening was used to further analyze the structure and function of VP1. The epitope information obtained in this study will be used to establish serodiagnostics for PPV infection and possibly to develop a vaccine.

## Materials and methods

### Virus and cells

PPV BQ strain was a field isolate identified by sequencing, and its complete genome sequence was submitted to GenBank with accession no. EU790641[17]. SP2/0 cells were kept in the Harbin Veterinary Institute (HVRI) of Chinese Academy of Agricultural Sciences.

### Preparation and purification of monoclonal antibodies (mAbs)

BALB/c female mice, obtained from the Experimental Animal Center of HVRI, were intraperitoneally immunized with 100 mg/ml of PPV, which was purified, dissolved in PBS, and mixed with MONTANIDETM IMS 1312 adjuvant (virus in PBS:adjuvant at 1:1). The adjuvant solution was kindly provided by the SEPPIC Company. Mice were later inoculated every 2 weeks for four times total. Seven days after the last booster inoculation, peripheral blood was collected from the tail vein of each mouse and was analyzed by indirect ELISA as described in Sulkanen et al. [18]. The mouse with the highest titre of anti-PPV antibodies was chosen for the fusion protocol. mAbs were derived by somatic cell hybridization of SP2/0 myeloma cells to spleen cells from the selected mouse as described by Kohler and Milstein [19] with minor modifications. Cell culture supernatants were screened by indirect ELISA using plates coated with activated PPV. Positive cell cultures were expanded and cloned by limit dilution. After three rounds of selection, 15 mAbs were generated following conventional protocols. The 15 mAbs did not react to Porcine Circovirus, Porcine Pseudorabies Virus, or Porcine Reproductive or Respiratory Syndrome Virus (data not shown). The 15 mAbs belong to the IgG1 subclass, and they have κ type light chains. The ascites titres of these mAbs were determined.

#### Western blotting assay

Western blot was performed according to standard procedure. Purified protein samples (the recombinant pET-VP1) were separated by SDS-PAGE with 12% gel prior to electrophoretic transfer to a nitrocellulose membrane. Western transfer was carried out in cold transferring buffer (0.025 M Tris-0.19 M glycine, 20% methanoland). Nitrocellulose membrane was then blocked overnight at 4°C with 10% skimmed milk in TBST (Tris-buffered saline with 0.1% Tween 20, pH 8.0). After washing with TBST, the membrane was incubated for 60 min with mAbs at 4°C. The membrane was washed and incubated for 60 min with horseradish peroxidase-conjugated rabbit anti-pig antibody. After further washing, immunoreactive proteins were visualized using DAB.

##### IFA

The IFA was carried out using fixed 96-well cell culture plate, the mAbs were diluted 1:20 in PBS (pH7.2). 96-well cell culture plate were equilibrated to room temperature and 50 μl of the diluted mAbs was placed in one well; each slide was incubated with a positive and a negative control serum diluted 1:20. The slides were incubated in a humidified chamber at 37°C for 30 min, flushed with PBS, then soaked for 10 min in PBS and blotted. Fifty microliter of a 1:200 dilution of fluoresce in isothiocyanate-labeled goat anti-mice IgG was added to each well and the slides were incubated at 37°C for 30 min and then washed as before. The slides were dried and a coverslip mounted with one drop of mounting fluid. Slides were examined for fluorescence immediately using a Zeiss fluorescent microscope at 400.

One of the mAbs, designated C4, was specific against VP1 of PPV (see Results). Ascites of C4 mAb were collected and purified by standard protein G column chromatography (Sigma) following the manufacturer's instructions.

### Library construction

The Ph.D.-12 phage display peptide library kit (New England Biolabs, Beverly, MA, USA) was used to screen for specific peptide binding to VP1 of PPV. The phage display library kit consists of a combinatorial library of random peptide 12-mers fused to a minor coat protein (pIII) of the M13 phage. The titer of the library is 1.5 × 10^13 ^pfu (plaque-forming units). The library contains a complexity of 2.7 × 10^9 ^individual clones, representing the entire obtainable repertoire of 12-mer peptide sequences, and each clone expresses a random 12-amino-acid sequence. Extensive sequencing of the naive library has revealed a wide diversity of sequences with no obvious positional biases. The *E. coli *host strain ER2738 (a robust F^+ ^strain with a rapid growth rate, New England Biolabs) was used for M13 phage propagation.

### Screening of phage display libraries

The phage libraries were screened by biopanning using standard methods with a few modifications [20]. Briefly, a 96-well microtitre plate was coated with 100 μg/ml of C4 in 0.1 M NaHCO_3 _buffer (pH 8.6) and incubated overnight at 4°C with gentle agitation in a humidified chamber. The coating solution was poured off, and each well was completely filled with blocking buffer (5 mg of bovine serum albumin [BSA]/ml in 0.1 M NaHCO_3 _buffer [pH 8.6]) and incubated for 1 h at 4°C. Protein-coated wells were washed with TBST (Tris-buffered saline plus 0.1% Tween 20) at least six times before biopanning of phage libraries. A Ph.D.-12 phage library with a titer of 10^11 ^pfu was incubated in the wells (100 μl/well) for 1 h at room temperature on a rocker. Nonbinding phage was poured off, and the wells were washed 10 times with 300 μL/well TBST. The phages binding to the anti-PPV-VP1 were then eluted with 0.2 M glycine-HCl buffer (pH 2.2) containing 1 mg BSA/ml. After elution, the acidic buffer was neutralized with 1 M Tris-HCl (pH 9.1). The eluted phage was then amplified *in vivo *on *E. coli *ER2738. The amplified phage was purified by double precipitation with 1/6 volume of polyethylene glycol (PEG)-NaCl (20% [vol/vol] PEG 8000, 2.5 M NaCl) and used in a second round of biopanning. In the first, second, and third round of biopanning, the Tween 20 concentration was increased progressively from 0.1 to 0.3 to 0.5% to wash off nonspecifically adhered phages. After three rounds of positive selection, *E. coli *ER2738 cells were infected with the eluted phage and grown on LB-agar plates coated with X-Gal (5-bromo-4-chloro-3-indolyl-ß-D-galactopyranoside at 0.04 m/v)/IPTG (0.05 m/v). Individual plaques were picked and amplified, and single-stranded phage DNA was isolated for sequencing.

### ELISA

ELISA using phage supernatant and purified antibody was used to evaluate the binding ability of individual phage clones. Briefly, the eluted phages from the third round of panning were transfected to *E. coli *and plated to LB/IPTG/Xgal plates. Following overnight incubation at 37 °C, blue plaques were randomly selected and amplified in *E. coli *in LB medium overnight at 37 °C. The phage supernatants from these infected *E. coli *were then used in the ELISA. Purified C4 mAb (100 μL/well in 0.1 M NaHCO_3 _buffer (pH 8.6)) was added to a 96-well microtiter plate and blocked with BSA (5 mg/ml). The selected peptide phage clones, amplified and concentrated by the PEG-NaCl precipitation method, were added to each coated well (10^10 ^pfu/well) and incubated for 1 h at 37°C. Unbound phages were removed by washing with TBST (0.5% Tween 20), and bound phage were detected with horseradish peroxidase-conjugated anti-M13 monoclonal antibody (Amersham Pharmacia Biotech, Uppsala, Sweden) at 1:5 000 and with TMB (3,3',5,5'-tetramethyl benzidine) substrate (Sigma). The optical density (OD) value of each well was read at 450 nm on an ELISA reader (Bio-Rad, Model 550). If the phage clones obtained after screening were true VP1 binders, then the ELISA signals should be substantially higher with the clones added than with the background.

### Competitive ELISA

Positive phage clones identified in the ELISA test were further explored for their specific binding to purified antibodies of PPV by a competition-inhibition ELISA. For each clone tested, PPV (1.0 μg/well) was mixed with phage (1 × 10^9 ^pfu/well) and added to the wells of a 96-well microtitre plate that had been previously coated with purified C4 (1.0 μg/well) as described above. The bound phage was detected as above using HRP/anti-M13 monoclonal antibody (1:5000). All tests were done in triplicate. One positive clone was selected for more detailed testing. This detailed testing involved a competitive ELISA, performed as described above except that serial dilutions of PPV were used.

### Sequence analysis of selected phages

After three rounds of *in vitro *panning, 50 plaques were randomly selected and their sequences analyzed with an ABI Automatic DNA Analyzer (Shanghai Bioengineering Ltd. Shanghai, China). The primer used for sequencing was 5'-CCCTCATAGTTAGCGTAACG-3'(-96 gIII sequencing primer, provided in the Ph.D.-12 Phage display peptide library kit, New England Biolabs). Homologous analysis and multiple sequence alignment were done using DNAStar and Clustal W programs to determine the relationships among the peptides.

### Mouse immunization and serum analysis

Sixteen 8-week-old female BALB/c mice were housed in the animal facility and allowed *ad libitum *access to feed and water. At day 0, the mice were divided randomly into four groups with four mice per group. Each mouse in group 1 was inoculated intramuscularly with with one of the 10 positive phage clones at a dose of 10^10 ^pfu [21]. The mice in group 2 were used as a negative control and were inoculated with the primer phage library at the same dose. The mice in group 3 were used as a positive control and were inoculated with 100 μL of PPV (100 μg/mL, buffered with PBS). The mice in group 4 were used as another negative control and were inoculated with 100 μL of PBS. All of the injections were mixed with the same volume of adjuvant (SEPPIC Company). A booster inoculation was carried out in the same manner for two times at 2-week intervals. At day 35, all the mice were killed by bleeding. The serum of each mouse was assayed by ELISA.

## Results

### Identification of mAb by western-blot and immunofluorescent antibody test

**Figure 1:**
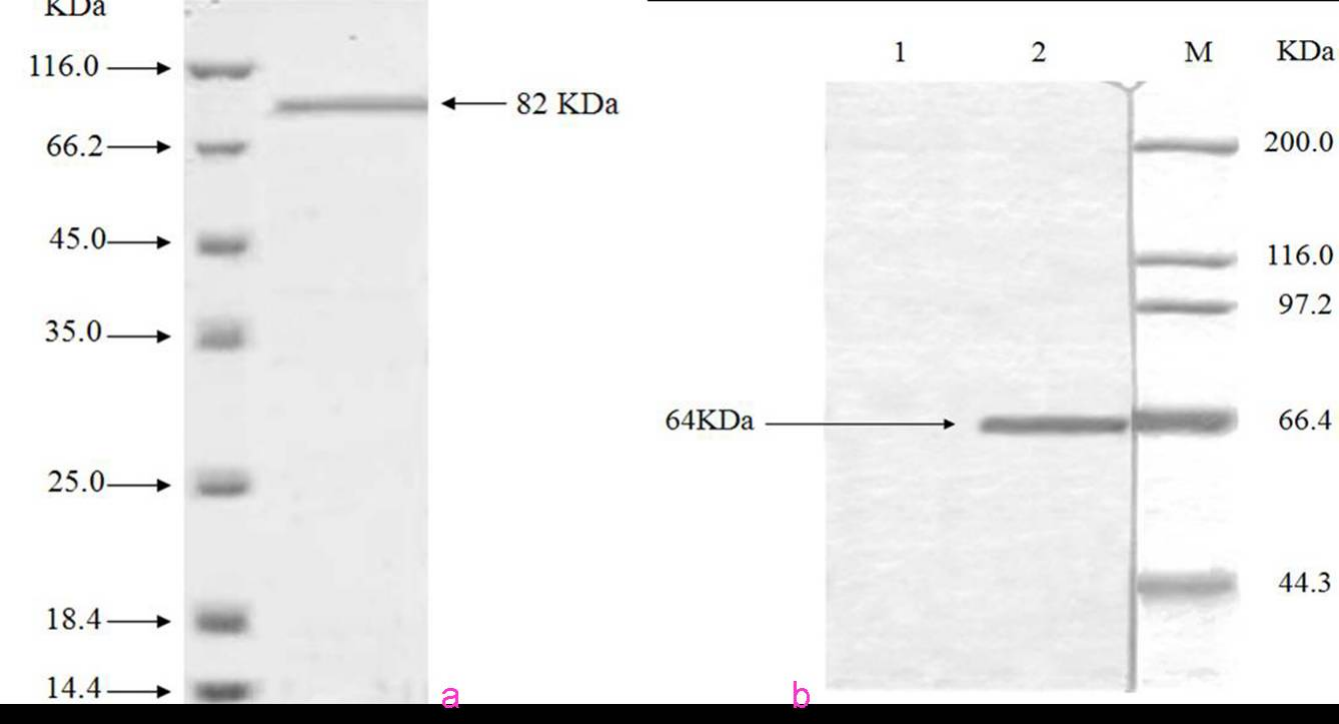
**Western-blot of mAbs with PPV**. a. Western-blot analysis of the purified mAb with PPV-VP1, 82 KD; b. Western-blot analysis of the purified mAb with PPV-VP2, 64 KD.

**Figure 2:**
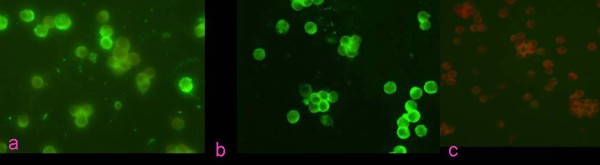
**The immunofluorescent antibody (IFA) test on ST cells**. a. IFA test of purified C4 reacted with PPV on ST cells; b. IFA test of positive sera reacted with PPV on ST cells; c. Negative control.

PPV was used as the antigen to produce specific mAbs. Fifteen hybridoma cell lines secreting antibodies against the VP1, VP2, and VP3 proteins were isolated. The mAbs were determined by western immunoblots (Fig 1a and Fig 1b). Two hybridoma lines were specific for protein VP1, and others were specific for VP2 or VP3. All of them also gave a good response in ELISA, with the titers of ascites ranging from 1:2 560 to 1:20 480. The mAb C4, which recognized VP1, was identified by the immunofluorescent antibody test (Fig 2), and it could produce high titer, then it was selected for biopanning.

### Peptide library screening

The B-cell epitopes of mAbs against the protein VP1 were studied by the phage display method. The affinity-purified antibodies were immobilized on the ELISA plate, and the bound phage clones were selected after three biopanning cycles. Ten positive phage clones were identified, and the ten selected phage clones produced by protein VP1 showed significant increases in reactivity to their antibodies (Table 1).

**Table 1:** The results and yield rate of three rounds of biopanning of the phage display peptide libraries.

Round	Input	Output	Yield (%)
1	1.5 × 10^11^	4 × 10^2^	2.7 × 10^-9^

2	1.5 × 10^11^	4 × 10^4^	2.7 × 10^-7^

3	1.5 × 10^11^	6 × 10^4^	4 × 10^-7^

### Homology analysis of exogenous sequences of selected phage clones

**Figure 3:**
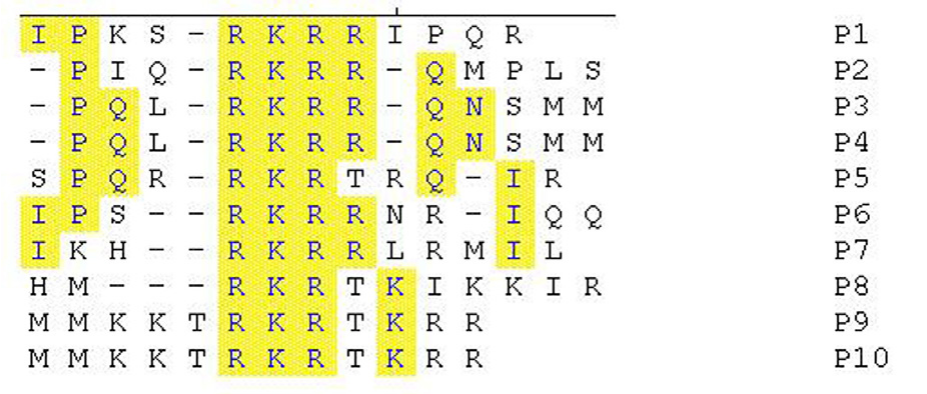
**Sequence comparison of the 10 positive phage clones**.

Ten positive phage clones highly reactive with antibodies to protein VP1 were further sequenced, and the corresponding exogenous sequence was given a sequential name from P1 to P10. The phage-displayed peptide sequences were aligned using DNAStar software to analyze the epitopes of the antibodies. The alignment analysis revealed amino acid homology in that all 10 clones contained a sequence of R-K-R (arginine-lysine-arginine). Amino acid sequences were identical for P3 and P4, and for P9 and P10. Clones P1 to P6 had a P residue on the RKR-N-terminal (Fig 3).

### Core identity and sequence analysis of the results

**Figure 4:**
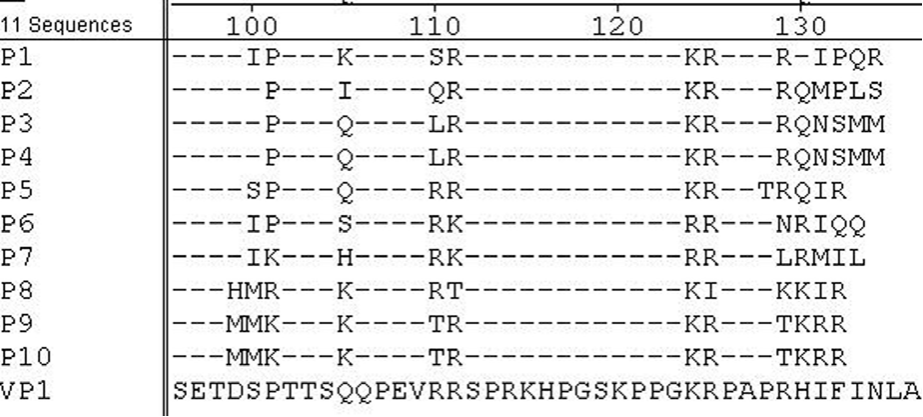
**Sequence comparison of the positive peptides and VP1-PPV BQ strain**.

Amino acid sequence homology between 10 positive clones and VP1 of PPV is shown in Fig 4. The results show that R-K-R is a mimotope motif of VP1 (Fig. 3). Sequencing showed that the 10 phage clones that reacted to the PPV mAb included eight different 12-peptide sequences, which all contained lysine and arginine as conserved residues but different amino acids in other positions. Except for clone P8, the other clones recognize the core sequence arginine-lysine-arginine.

### Mouse immunization and serum analysis

Anti-peptide sera colleted from mice immunized with the phage clones from the VP1 group were tested with HI and the competitive ELISA method to confirm the specificity of the polysera (inhibition by mAb). Each of the peptides was recognized by the respective serum from infected mice (Table 2). The BALB/c mice inoculated with positive clones produced antibodies that were recognized by both HI and competitive ELISA.

**Table 2:** Antibody titres in mice inoculated with positive phage clone.

Group	Titer of HI antibody	ELISA value
VP1 phage	2^8^	0.538

Negative	2^3^	0.129

Blank	2^2^	0.12

PPV	2^13^	1.212

## Discussion

To our knowledge, this is the first report on the use of phage display technology to screen epitopes or "mimotopes" of VP1 of PPV. VP1 and VP2 are different transcription products from the same gene. Although there is uncertainty about the exact length of VP1 because of the presence of different possible splicing sites, it might consist of up to 748 amino acid residues [22].

Identification of the VP1 epitope requires the preparation of an mAb. Such preparation includes selection and purification of the immunogen and determination of immunogenicity and immunogen dosage. A mAB C4 specific to VP1 of PPV was used as the target protein to screen the phage displayed peptide library. After three rounds of panning, phage clones capable of binding to the mAB C4 were enriched, showing an increase in titre. Although mAbs can be prepared with low-purity viral antigen, antigen impurity will affect the screening of mAbs and even influence the production of antibody. Because the virions of PPV are small, normal ultracentrifugation at 25,000-30,000 rpm and differential centrifugation are inadequate. In contrast, density gradient centrifugation is a simple and inexpensive technique that can effectively separate small proteins in different layers of the centrifugation medium. In the present study, we used cane sugar density gradient centrifugation to purify the immunogen. We found that the cane sugar solution should be used immediately after preparation because stored solutions can give poor results. The volume of viral solution added to the upper layer should not excel 3 ml; greater volumes can lead to gradient collapse. Antigen immunogenicity directly influences the quality of the mAb. Although artificially expressed protein has been used as the immunogen to prepare mAb in many studies, the structure of artificial protein can differ from that of natural protein, and we therefore selected the whole virus to inoculate the mice by intramuscularly. A preliminary study in our laboratory indicated that the inoculation dosage of 100 μg of viral solution per mouse, followed by two booster inoculations, resulted in the production of sufficient quantities of activated B cells that produced 15 high-affinity mAbs.

Indirect ELISA indicated that the 15 mAbs did not recognize other viruses (PCV2, PRV, and PRRSV). IFA showed that the 15 prepared strains all detected PPV. Western blot demonstrated that two of the strains specifically recognized VP1 protein (which is 82 KD), that 12 strains recognized both VP2 and VP3 (which are 64 KD), and that one strain was not imprinted. The latter strain might recognize the PPV conformation epitope, but further research is required to support that inference. Because VP2 is produced by VP3 hydrolysis, both have the same sequence region. We therefore conjecture that the 12 strains recognize the common antigen epitope of VP2 and VP3.

Phage random peptide display is a new technique for studying the epitope of simulated antigens. Before the current study, there were no reports on the use of phage display technology to identify epitopes of PPV antigens. Random phage peptide display was used to screen the PPV-VP1 simulated antigen epitope, and bioinformation analysis indicated that the β-turn and random coil of the screened epitope region mainly lies on the protein surface. We used DNAstar software analysis of the secondary structure of matured multi-peptide related to the simulated antigen epitope sequence to determine that arginine-lysine-argine probably form the antigen epitope.

The results indicate that the sequence arginine-lysine-argine is the key component of the epitope of the simulated PPV-VP1 antigen. Whether a simulated antigen with this epitope can substitute for VP1 protein antigen requires further immunogenicity studies with animals. To verify whether the positive phage clones from the random peptide phage library can induce the production of specific antibodies, the phage clones were inoculated into BALB/c mice, and the specific serum antibody titers in the mice were determined. The results showed that BALB/c mice inoculated with positive clones produce antibodies that were detected by both HI and ELISA. The mouse immunization trial therefore provides strong evidence that the mimotope played a role in producing antibody against PPV. This further illustrates that the 12-bit analog form does induce antibodies. The peptides will of course be recognized by the corresponding sera from infected mice, even they are not specific. In addition, to confirm the importance of AKR motif, control peptides containing no or mutated AKR motif need be synthesized and used as antigen to test sera samples from infected mice and naturally infected pigs.

## Conclusion

In summary, the current study has provided new information about the structure of VP1 in PPV, information that will be useful for further research on gene structure and function of VP1. The study also shows the potential for the use of peptide mimotopes as alternatives that are currently used for diagnosis of PPV infection and for PPV vaccines.

## Competing interests

The authors declare that they have no competing interests.

## Authors' contributions

HX, ZW carried out the experiments and wrote the manuscript. SC and YC conceived the studies and participated in experimental design and coordination. All authors read and approved the final manuscript.
